# A Systematic Review of Drone Customization and Application in Public Health Innovation

**DOI:** 10.7759/cureus.85435

**Published:** 2025-06-05

**Authors:** Sudip Bhattacharya, Alok Singh, Shailesh Tripathi, Poonam Kushwaha, Himel Mondal, Vanisree Ramanathan, Deep Sikha, Shiv K Mudgal, Samiksha Bhattacharjee, Sandip Bhattacharya, Akanksha Singh, Vimal S Munda, Keerti B Pradhan

**Affiliations:** 1 Community and Family Medicine, All India Institute of Medical Sciences, Deoghar, IND; 2 Community Medicine, Shree Guru Gobind Singh Tricentenary University, Gurugram, IND; 3 Naturopathy and Yogic Sciences, Shree Guru Gobind Singh Tricentenary University, Gurugram, IND; 4 Hospital Administration, Rajendra Institute of Medical Sciences, Ranchi, IND; 5 Community Medicine, Rama Medical College Hospital and Research Centre, Kanpur, IND; 6 Physiology, All India Institute of Medical Sciences, Jharkhand, IND; 7 Public Health, Dr. Vishwanath Karad MIT World Peace University, Pune, IND; 8 Community Medicine, Himalayan Institute of Medical Sciences, Dehradun, IND; 9 College of Nursing, All India Institute of Medical Sciences, Deoghar, IND; 10 Pharmacology, All India Institute of Medical Sciences, Deoghar, IND; 11 Electronics and Communication Engineering, Sri Rajeshwara University, Warangal, IND; 12 Ayurvedic Medicine, Mahatma Gandhi Kashi Vidyapith University, Varanasi, IND; 13 Microbiology, All India Institute of Medical Sciences, Deoghar, IND; 14 Chitkara Business School, Chitkara University, Chandigarh, IND

**Keywords:** drone, emergency medical service, healthcare service delivery, public health, unmanned aerial vehicle

## Abstract

A drone, also known as an unmanned aerial vehicle (UAV), is an aircraft operated without a human pilot on board embedded with diverse capabilities. Recent advancements in drone technology have expanded their applicability beyond military and recreational use, enabling critical functions across civilian sectors. In particular, the integration of UAVs into healthcare systems presents new opportunities to overcome logistical barriers, especially in hard-to-reach or underserved regions. This review aims to explore the expanding horizon of drone services within the public health domain and identify future research avenues in this innovative field. In line with the Preferred Reporting Items for Systematic Reviews and Meta-Analyses guidelines, this systematic review followed a structured approach to screen and select studies. Initially, a comprehensive search was conducted across five major databases: PubMed, Google Scholar, IEEE Xplore, Web of Science, and Scopus. Search terms included combinations of “unmanned aerial vehicle,” “drone,” “UAV” with “public health,” “healthcare delivery,” “medical logistics,” “telemedicine,” and “remote healthcare.” Boolean operators and search filters were applied to refine results, yielding an initial count of 1,200 articles from which only 36 have met our inclusion criteria. We found that drones are capable of serving in multiple arenas such as pharmaceutical delivery, blood and organ transportation, disaster response, aerial telemedicine stations, environmental monitoring, remote diagnostics, patient monitoring and care, training and simulation, nutritional support, water and sanitation, psychological support, bio medical waste management, public health surveillance, veterinary care, mobile laboratory services, support of elderly care and education and outreach.

## Introduction and background

A drone, also known as an unmanned aerial vehicle (UAV), is an aircraft operated without a human pilot on board. Instead, drones are typically controlled remotely by a human operator or autonomously through pre-programmed flight plans or onboard sensors and navigation systems [1]. Drones come in various sizes and shapes, from small quadcopters used for recreational purposes to larger fixed-wing aircraft employed for military reconnaissance or commercial applications. They are equipped with cameras, sensors, and sometimes additional payloads such as scientific instruments or cargo compartments, enabling diverse functionalities such as aerial photography, surveillance, delivery of goods, disaster response, and environmental monitoring [2]. Drones have revolutionized industries by offering cost-effective, efficient solutions for tasks that were once difficult or dangerous to perform manually or with traditional aircraft. Their versatility and adaptability continue to expand as technology advances, making drones integral to modern applications across various sectors worldwide [3,4].

Drones are increasingly being utilized in the healthcare sector to enhance the efficiency, accessibility, and speed of medical services [5]. They are particularly valuable for transporting medical supplies, such as blood products, vaccines, medications, and diagnostic samples, to remote or disaster-stricken areas where traditional transport methods may be delayed or impossible [6]. In emergency situations, drones can deliver automated external defibrillators (AEDs) or first aid kits to patients before medical teams arrive [7]. Additionally, drones are used for surveillance during disease outbreaks, helping in mapping affected areas and supporting public health interventions. Their ability to bypass traffic and geographical barriers makes them a promising tool for improving healthcare delivery, especially in underserved regions [8]. Hence, these technological advances are particularly relevant in the context of global healthcare inaccessibility, which remains a critical barrier to achieving equitable health outcomes.

With this background, our aim in this paper is to explore the expanding horizon of drone services within the public health domain, as well as to identify future research avenues in this innovative field.

## Review

Methods

In line with the Preferred Reporting Items for Systematic Reviews and Meta-Analyses (PRISMA) guidelines, this systematic review followed a structured approach to screen and select studies. Initially, a comprehensive search was conducted across five major databases: PubMed, Google Scholar, IEEE Xplore, Web of Science, and Scopus. Search terms included combinations of “unmanned aerial vehicle,” “drone,” “UAV” with “public health,” “healthcare delivery,” “medical logistics,” “telemedicine,” and “remote healthcare.” Any study in English discussing the use of drones in healthcare or related fields was included. Eligible study designs included peer-reviewed randomized controlled trials, cohort, case-control, observational studies, and reviews. We excluded studies involving drones used outside healthcare, such as in military or agriculture, and those mentioning drones without relevant health applications.

**Figure 1:**
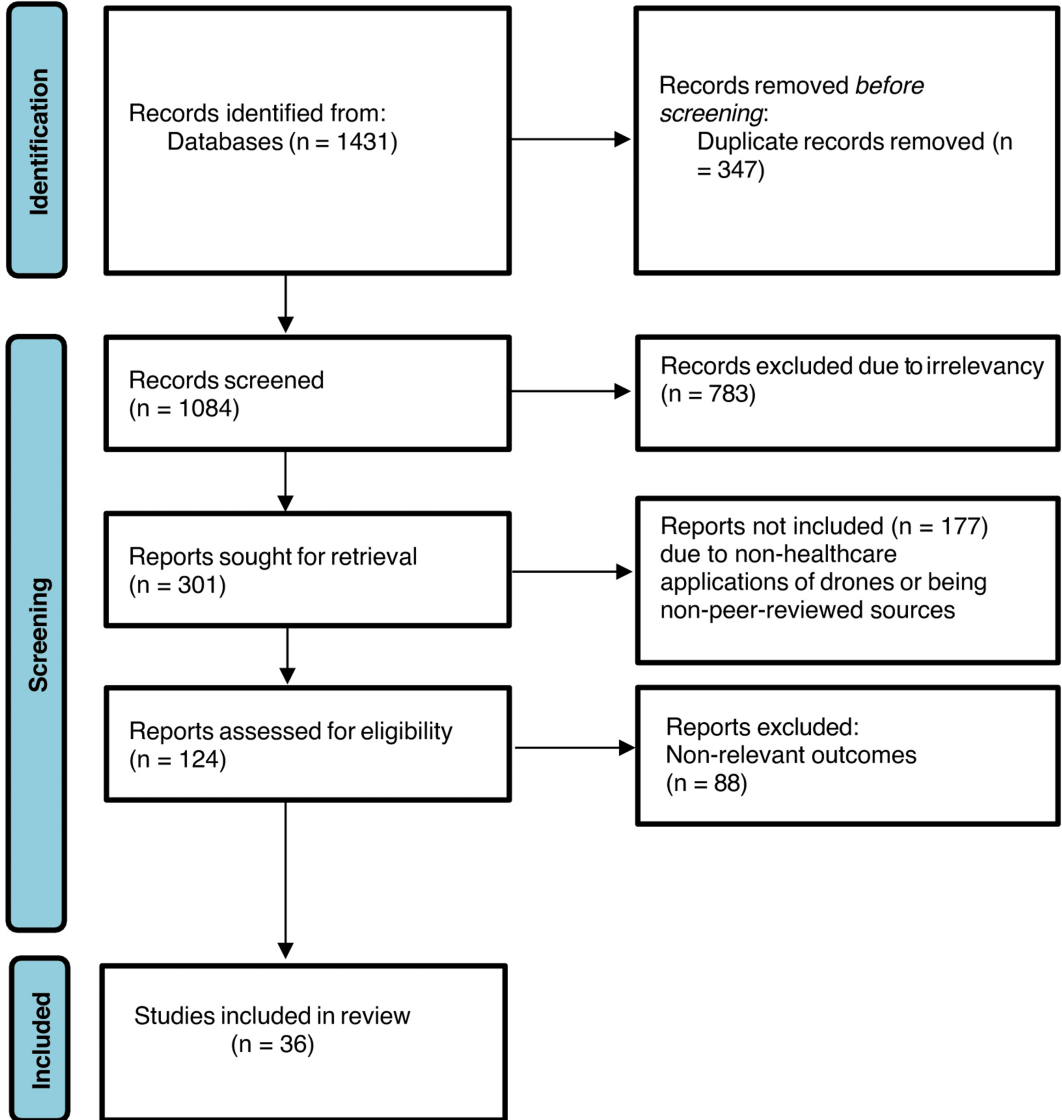
Preferred Reporting Items for Systematic Reviews and Meta-Analyses (PRISMA) flow chart n: number

A total of 1431 articles were found from the database, and finally, 36 articles were included in the systematic review. The study number in the different screening phases is shown in Figure 1.

Result and discussion

The applications of drones in various healthcare sectors are summarized in Table 1 and discussed below. 

**Table 1:** Articles related to application of drone in helthcare

Author	Year	Healthcare application
Hashim [1]	2025	Enable terrestrial wireless communications by forming an assisted communication system and acting as aerial base stations or communication access points
Peksa & Mamchur [2]	2024	Aerial photography and videography, surveillance and security, mapping and surveying, search and rescue, educational and research purposes
Agarwalla et al. [5]	2025	Disaster Relief, medical transport, and telemedicine
Aggarwal et al. [6]	2023	Delivery of medical supplies
Schierbeck et al. [7]	2023	Delivery of automated external defibrillators for cardiac arrest management
Olatunji et al. [8]	2023	Delivery of vaccines, medications, blood samples, diagnostic tools
Bhattacharya et al. [9]	2023	Emergency medicine, management of cardiac arrest by automated external defibrillators
Bhattacharya et al. [10]	2020	Transportation of blood, medicines, and biologicals, public health surveillance
Stephan et al. [11]	2022	Delivery of medical supplies
Beck et al. [12]	2020	Delivery of adrenaline auto-injectors for anaphylaxis
Griffith et al. [13]	2023	Delivering rift valley fever vaccines
Dukkanci et al. [14]	2023	Delivering relief logistics in earthquake
Sharma & Sharma [15]	2024	Logistic supply in healthcare
Emad Alfaris et al. [16]	2024	Delivering humanitarian relief material in disasters
Knoblauch et al. [17]	2025	Supply chain management in bringing essential medicines and health commodities
Nyaaba & Ayamga [18]	2021	Delivering blood, drugs, vaccines, and laboratory test samples
Sigari & Biberthaler [19]	2021	Telemedical applications, optimized emergency medical services, laboratory transportation
Stierlin et al. [20]	2024	Medical supply transportation
Scalea et al. [21]	2021	Delivery of human organ for transplantation
Nooralishahi et al. [22]	2021	Inspection of industrial sites for health safety check of workers
Rejeb et al. [23]	2021	Search and rescue tasks in humanitarian crisis, providing an instant telecommunications infrastructure, delivery of emergency supplies, search and rescue activities, and mapping of disaster regions.
Partheepan et al. [24]	2023	Bushfire management and search and rescue operation
Nedelea et al. [25]	2022	Telemedicine system applicability using drones in pandemic emergency medical situations
Bayomi & Fernandez [26]	2023	Climate change mitigation for healthy environment
Georgiou et al. [27]	2022	Criminology and legal use
Al-Wathinani et al. [28]	2023	Prehospital care in emergency like - carrying medical equipment, including defibrillators, oxygen tanks, and medications
Mechan et al. [29]	2023	Vector control for malaria
Gutsa et al. [30]	2024	Monitoring debris accumulation hotspot for water sanitation
Ayamga et al. [31]	2021	Surveying, humanitarian work, disaster risk management, research, and transportation
Fartook et al. [32]	2024	Emotional support
Mohan et al. [33]	2022	Remote patient monitoring
Sliusar et al. [34]	2022	Municipal solid waste management and landfilling
Rayner et al. [35]	2024	Animal health management
Comtet et al. [36]	2022	Transporting laboratory samples
Balasingam [37]	2017	Assisting elderly care
Johal et al. [38]	2022	In education (social drones)

Pharmaceutical Delivery

Customizing drones with secure compartments for pharmaceutical and medical supply delivery involves designing specialized containers to ensure the integrity and safety of medications during transport. These compartments maintain the efficacy of temperature-sensitive medications and vaccines during delivery to healthcare facilities, pharmacies, or patients in remote areas. They feature insulated materials and temperature regulation systems to control the internal climate, shock-absorbing mechanisms to protect fragile items, and tamper-proof seals and locking mechanisms for security. Airtight and waterproof seals safeguard medications from moisture and contaminants. Additionally, custom compartments may include organization features and remote monitoring capabilities for efficient storage and immediate response to any deviations. This customization enhances the reliability and efficiency of medical supply chains, enabling timely and secure delivery where traditional methods may be impractical [11]. At present, there is no unified standard set by aviation authorities or standardization bodies (such as the International Civil Aviation Organization (ICAO) Standards and Recommended Practices) concerning the specifications for containers used in the transport of potentially hazardous materials via drones. To ensure an effective design, both the integrity of the product and the safety of third parties must be taken into account, though the specific requirements can differ based on the nature of the substance and the form in which it is transported [12].

For example, Zipline (Zipline International Inc., South San Francisco, CA, USA) operates one of the largest drone delivery networks globally, delivering blood products, vaccines, and essential medications to remote areas in Rwanda, Ghana, and other countries [10]. Zipline drones flew distances of up to 80 km and carried payloads of up to 2 kg. The Ministry of Health in Rwanda paid a flat monthly rate that covered unlimited drone flights for Rift Valley Fever (RVF) vaccine delivery. At the time, drones were only launched and landed at two distribution centres, which made it impossible to transport items from the field. The company has extended its services to 350 medical facilities [13].

Since its inception in Rwanda in 2016, Zipline has expanded its operations to several other countries, including Ghana, Nigeria, and the United States. In Rwanda alone, Zipline's drones completed over 200,000 deliveries, covering more than 2 million kilometres (approximately 1.2 million miles) to transport blood, vaccines, and essential medications to remote healthcare facilities. This service significantly reduced the delivery time for critical supplies, which previously took hours or even days by road, to just 30 minutes by drone. The service has had a profound impact, especially in emergencies where the rapid delivery of blood has been instrumental in saving numerous lives [14,39].

Similarly, UPS Flight Forward (UPS Flight Forward Inc., United Parcel Service, Inc., Atlanta, GA, USA) has partnered with CVS to deliver prescription medications to residents of The Villages, Florida, using drones to transport drugs directly to customers' homes [40]. Swoop Aero (Kite Aero, Sydney, Australia) operates in Malawi, focusing on delivering essential medical supplies to remote clinics and communities [41]. Swoop Aero drones have delivered over 500,000 vaccines and medical supplies to remote communities, significantly enhancing healthcare access. With a capacity of up to 3 kilograms and a range of 130 kilometres per flight, these drones have been crucial in boosting vaccination rates and improving health outcomes in areas with limited transportation options. This success underscores the vital role drones play in addressing logistical challenges in global health [42].

DHL (DHL, San Francisco, CA, USA) has conducted trials of its Parcelcopter with Wingcopter in Germany and Tanzania, delivering medications and vaccines to remote islands and rural areas [43]. Matternet project in Zurich, Switzerland, collaborates with healthcare partners to transport medical samples and supplies between hospitals and laboratories. The operation between hospitals in Zurich reduced transportation times from hours by road to just seven minutes by drone [20].

Blood and Organ Transportation

Drones are increasingly customized for transporting blood for transfusions and organs for transplants, revolutionizing medical logistics with their speed and precision. These specialized drones feature state-of-the-art containers designed to preserve blood and organs optimally throughout their journey. Blood containers maintain specific temperature ranges to prevent clotting and degradation, using insulated compartments and cooling mechanisms. Similarly, organ containers provide a controlled environment with sensors for temperature, humidity, and real-time data transmission to ensure continuous oversight. This careful management facilitates rapid and reliable delivery, overcoming geographical barriers and significantly reducing transportation times compared to traditional methods. This capability is crucial in emergencies where timely access to blood or organs can mean the difference between life and death [3,4].

For instance, LifeDrone by MissionGO (MissionGO, Inc., Baltimore, MD, USA), in collaboration with the Nevada Donor Network (Las Vegas, NV, USA), conducted the first-ever organ delivery via drone in the U.S., transporting a donor kidney over 10 miles from an airport to a location outside a small town in Las Vegas [44]. Similarly, UPS and Matternet have collaborated to transport medical samples and supplies between hospitals in North Carolina and Switzerland, showcasing the potential of drones in medical logistics [21]. These examples highlight the ongoing efforts and successful demonstrations of using drones for blood and organ transportation, underscoring their potential to revolutionize emergency medical logistics and improve patient outcomes worldwide.

Disaster Response

Drones customized for disaster response provide rapid and effective assistance in the aftermath of natural disasters or humanitarian crises, where traditional infrastructure may be damaged or inaccessible. These drones carry emergency medical supplies, search and rescue equipment like thermal imaging cameras, and sensors to detect signs of life under debris. They also relay real-time video feeds and data to emergency responders, enhancing situational awareness and coordination. Their ability to navigate disaster zones swiftly enables timely assistance in locating and rescuing survivors, delivering critical supplies, and providing essential situational updates. Examples include the DJI (Shenzhen, China) Matrice 300 RTK, used globally by emergency responders and equipped with thermal cameras and high-resolution sensors to assess disaster areas, locate survivors, and identify hazards [22,45]. The Red Cross is testing drones to deliver emergency supplies like medical kits and communication devices to disaster-stricken areas efficiently [23,46]. United Nations Children's Fund (UNICEF) conducted trials in Vanuatu to survey damage and deliver supplies to communities isolated by Cyclone Pam, demonstrating drones' potential in humanitarian aid [47]. Australian firefighting agencies tested drones with infrared cameras and fire-retardant delivery systems to assist in wildfire response, providing real-time aerial intelligence [24]. GlobalMedic’s (GlobalMedic, Toronto, Canada) RescUAV initiative can be used for rapid damage assessment, delivering emergency supplies, and supporting search-and-rescue operations [48]. These examples showcase the diverse applications of drones in disaster response, improving the efficiency, safety, and effectiveness of relief efforts by providing valuable aerial capabilities that complement traditional methods.

Aerial Telemedicine Stations

Aerial telemedicine stations use large drones as mobile healthcare units equipped with advanced medical tools like examination kits and ultrasound devices, allowing healthcare professionals to provide treatments directly in remote or disaster-stricken areas. These drones enable real-time remote consultations via integrated telecommunication systems, crucial in scenarios where traditional healthcare infrastructure is compromised [25]. These initiatives demonstrate the potential of drones to revolutionize emergency medical response and improve healthcare access in challenging environments.

Environmental Monitoring

Customizing drones with specialized sensors to monitor environmental factors advances proactive healthcare management. These drones detect air quality, radiation levels, and pathogens, providing real-time data to authorities for timely preventive measures. Examples include NASA's Global Hawk drones for atmospheric research, WWF's use of drones to monitor wildlife and habitats, marine biologists studying ecosystems, forestry agencies detecting illegal logging, and environmental agencies assessing water quality [26]. Drones enhance environmental monitoring by offering aerial perspectives, real-time data, and access to remote areas, supporting conservation, climate research, and resource management.

Training and Simulation

Healthcare professionals have a positive attitude towards training on drones in healthcare [49]. Drones customized for medical education and training programs are innovative tools that simulate essential scenarios to prepare healthcare professionals effectively. They replicate emergencies like cardiac arrest and trauma injuries, allowing practice in timely interventions and decision-making under pressure. In surgical training, drones simulate procedures with precision, offering practice in techniques and instrument handling [50]. For disaster response drills, they simulate mass casualty incidents and infectious disease outbreaks, facilitating rehearsal of responses and triage protocols. These drones may integrate high-resolution cameras, sensors for real-time data feedback, simulation software, and virtual reality (VR) technology to create immersive environments. They provide a safe and realistic platform for refining critical skills, improving teamwork, and familiarizing oneself with emergency protocols without live scenario risks. Ultimately, they enhance healthcare competence and readiness, ensuring professionals deliver high-quality care and respond effectively to diverse medical challenges [19].

For instance, military forces use drones for reconnaissance and surveillance training, while law enforcement agencies employ drones for search and rescue operations and crime scene investigations [51]. Fire departments utilize drones with thermal cameras for firefighting simulations and structural assessments, enhancing training in hazardous environments [24]. Medical schools and hospitals use drones equipped with surgical tools for training in minimally invasive procedures and emergency responses in remote areas [10]. These can potentially be used in training staff.

Remote Diagnostics

Customizing drones with medical imaging devices, tumor scanner equipment enables them to perform essential diagnostics in remote or underserved areas lacking traditional medical facilities [52]. These drones can carry compact, portable imaging technologies for on-site scans of internal organs or skeletal tissues. Real-time transmission of data and images via secure telecommunications allows remote specialists to promptly interpret scans, guiding timely medical interventions, treatment plans, or referrals by local healthcare providers. This capability is crucial in emergencies, rural settings, disaster responses, or during disease outbreaks, ensuring rapid diagnosis and improving patient outcomes. For instance, FlyPulse (FlyPulse, Trollhättan, Sweden) has developed a tele-medical drone equipped with cameras and sensors to support remote medical assessments and vital sign monitoring in emergencies [53].

Patient Monitoring and Care

Customizing drones with sensors and cameras for remote patient monitoring is a significant advancement in healthcare, especially for rural or home care settings. These drones monitor vital signs like blood pressure, heart rate, and blood glucose levels using integrated sensors, transmitting real-time data to healthcare providers for continuous monitoring and early detection of health changes [8]. In post-operative care, drones with cameras can assess wound healing and monitor for complications remotely [33], reducing the need for patient travel. This technology improves healthcare efficiency by minimizing in-person visits while ensuring timely medical attention and support, enhancing management of chronic conditions and post-operative care, and ultimately improving patient outcomes.

Water and Sanitation

Drones equipped with water purification systems and sanitation supplies play a vital role in delivering essential humanitarian aid to disaster-stricken or remote areas. These specialized drones feature compact purification systems that treat contaminated water from sources like rivers or ponds, ensuring safe drinking water directly reaches affected communities during emergencies [54]. They also distribute hygiene kits with soap and disinfectants to prevent waterborne diseases and improve hygiene practices. Drones can conduct aerial spraying to disinfect large water bodies and sanitation facilities, reducing disease transmission risks. Their agility enables quick deployment to inaccessible areas, overcoming logistical challenges in traditional relief efforts. By providing immediate access to clean water and sanitation, these drones support humanitarian relief operations, enhance emergency response capabilities, and improve conditions for vulnerable populations globally [28].

For example, DJI drone was used to monitor water quality in lakes [55], rivers, and coastal areas for environmental agencies. WaterDrone specializes in water sampling and quality monitoring, detecting contaminants like temperature and dissolved oxygen levels [56].

Nutritional Support

Customized drones for nutritional aid delivery revolutionize food security efforts in remote or inaccessible regions. These drones are equipped with specialized compartments that include insulated containers and climate control systems to ensure the freshness and nutritional quality of food supplies during transport. They carry a variety of essential nutritional items such as vitamin-enriched supplements, ready-to-eat packets, and fortified foods containing proteins, vitamins, and minerals, or edible wings [57]. This technology is particularly critical in humanitarian crises, natural disasters, or conflict zones where conventional food distribution is hindered. By utilizing drones, humanitarian organizations can rapidly deploy nutritional aid, circumventing infrastructure challenges and logistical delays. Drones provide a cost-effective and efficient solution for delivering timely nutritional support, mitigating food insecurity, and related health risks linked to malnutrition. Their capability to navigate challenging terrain ensures equitable distribution and direct delivery to affected populations, thereby enhancing access to vital food resources. Customized drones significantly improve food access, nutrition outcomes, and overall well-being among vulnerable communities, underscoring their pivotal role as innovative tools in global efforts to combat hunger and malnutrition [31].

Psychological Support

Customized drones with communication and counselling capabilities provide remote psychological support and mental health services to isolated or disaster-affected communities. Equipped with telecommunication systems for real-time video conferencing and audio communication, these drones enable licensed mental health professionals to conduct counselling sessions with individuals or groups. They also deliver educational materials and coping strategies via videos, pamphlets, or interactive modules to help manage stress and trauma during crises [32]. Drones' mobility allows them to swiftly reach remote areas where traditional mental health services are limited, making them invaluable in disaster response situations. By fostering supportive environments and promoting mental well-being, these drones enhance community resilience and reduce stigma associated with seeking mental health care. Leveraging technology for mental health delivery, customized drones improve access to critical support services and contribute to overall community resilience during challenging circumstances like any disease pandemic [58].

Public Health Surveillance

Customizing drones with specialized sensors for monitoring vector-borne diseases like malaria is a significant advancement in public health surveillance and disease prevention [29]. Equipped with thermal imaging cameras, hyperspectral sensors, and environmental sampling devices, these drones gather comprehensive data on factors conducive to disease transmission. For instance, they survey mosquito breeding grounds, monitor vegetation density, and assess water quality in potential disease hotspots. The real-time transmission of data to public health authorities allows for rapid analysis and interpretation, facilitating early detection of disease outbreaks. This early warning system supports targeted interventions such as larviciding campaigns, mosquito control measures, and community awareness initiatives. Drones also enable spatial mapping [59], and they can be used for predictive modelling of disease dynamics, integrating geospatial data with epidemiological information to identify high-risk areas and allocate resources effectively. Drones' agility and mobility enable access to remote or inaccessible locations, enhancing disease surveillance capabilities where traditional methods fall short. By providing timely environmental data, customized drones improve disease surveillance, early outbreak detection, and ultimately reduce the impact of vector-borne diseases on communities. This innovative use of drone technology underscores its potential as a valuable tool in global health efforts to combat infectious diseases and safeguard public health worldwide [10,17,23].

Biomedical Waste Management

Drones are customized to transport medical waste from remote clinics or disaster sites to central disposal facilities, addressing biohazardous material management challenges and ensuring safe disposal. Equipped with secure containers, these drones handle various types of medical waste, including contaminated supplies and infectious materials, preventing leakage or exposure with features like double-layered seals and puncture-resistant materials [34]. Some drones also integrate sterilization equipment, such as autoclaves, to neutralize pathogens during transit. Their mobility allows them to reach inaccessible areas swiftly, supporting proper disposal according to biohazard waste regulations. This application enhances safety, reduces environmental impact, and promotes responsible biohazard management in healthcare and emergency settings [60].

Veterinary Care

Drones can support veterinary services by delivering medications, vaccines, or diagnostic tools to livestock in remote rural areas. They can also assist in monitoring animal health and tracking disease outbreaks among livestock populations [61].

Mobile Laboratory Services

Drones as mobile laboratories equipped with diagnostic testing gear revolutionize disease surveillance and environmental monitoring. These specialized drones feature portable, lab-grade equipment for rapid on-site analysis of infectious diseases or environmental pollutants. They employ molecular diagnostic tools like polymerase chain reaction (PCR) machines or immunoassay kits to detect pathogens or contaminants in samples collected directly from affected areas. Real-time analysis within the drone provides immediate diagnostic data crucial for early disease outbreak detection, environmental monitoring, or disaster zone assessment. Wireless transmission of results enables prompt decision-making by healthcare and environmental authorities, facilitating swift interventions tailored to disease control, environmental cleanup, or targeted surveillance efforts [36].

Support for Elderly Care

Customized drones offer innovative solutions to support elderly individuals, enhancing their independence and quality of life, especially in remote or independent living situations. These drones deliver medication reminders, prescriptions, and essential medical devices directly to seniors, ensuring medication adherence and accessibility to necessary healthcare tools. They also serve as emergency response systems, enabling real-time communication for immediate assistance during medical emergencies. By addressing these needs effectively, drones contribute significantly to revolutionizing elderly care, leveraging advanced technology to meet the specific challenges of aging populations globally [37].

Education and Outreach

Customized drones are pivotal for health education and community outreach, enhancing health literacy and empowering underserved populations. These drones deliver diverse educational materials directly to remote or isolated areas, including brochures, videos, and health messages, overcoming geographic barriers. They provide culturally relevant content and facilitate interactive workshops and virtual consultations with healthcare professionals, fostering engagement and promoting healthier behaviors [38]. By improving access to healthcare information and promoting proactive health management, drones play a transformative role in enhancing community well-being and resilience worldwide.

Strengths and limitations

This systematic review presents an overview of drone customization and application in public health, highlighting their versatile roles in healthcare delivery, emergency response, diagnostics, surveillance, and outreach. The strength of the review lies in its broad scope, encompassing both conventional and innovative uses of drones, supported by real-world examples from diverse geographical settings, which enhances its global relevance. However, the review is limited by the absence of a detailed quality assessment of the included studies, which may influence the strength of the conclusions drawn. Moreover, the discussion predominantly focuses on descriptive aspects, with limited analysis of cost-effectiveness, regulatory considerations, and long-term sustainability, factors that are critical for informing policy and practical implementation.

## Conclusions

This review underscores the transformative potential of drone technology in healthcare logistics, emphasizing the need for continued research and investment in this field. The specific applications demonstrated in our analysis highlight how drones can improve the efficiency, safety, and efficacy of medical supply chains, disaster response efforts, and public health initiatives. Future research should focus on further refining drone technologies and exploring their expanded applications within diverse healthcare contexts to maximize their impact on global health outcomes.
